# Global, regional, and national burden of spinal injuries attributable to road injuries: a systematic analysis of incidence, prevalence, and YLDs with projections to 2046

**DOI:** 10.3389/fpubh.2025.1628455

**Published:** 2025-09-18

**Authors:** Yanqing Deng, Yansong Feng, Xuancheng Ou, Yanni Lan, Jiyong Wei, Qing He

**Affiliations:** ^1^Department of Spine Surgery, The Central Hospital of Yongzhou, Yongzhou, Hunan, China; ^2^Department of Spine Surgery, Yongzhou Hospital Affiliated to University of South China, Yongzhou, Hunan, China; ^3^Department of Orthopaedics & Traumatology, Fourth Affiliated Hospital of Guangxi Medical University/Liuzhou Worker's Hospital, Liuzhou, Guangxi, China; ^4^Department of Pharmacy, The People's Hospital of Guangxi Zhuang Autonomous Region & Guangxi Academy of Medical Sciences, Nanning, Guangxi, China; ^5^Department of Orthopedic Surgery, The First People's Hospital of Nanning, The Fifth Affiliated Hospital of Guangxi Medical University, Nanning, Guangxi, China

**Keywords:** spinal injuries, road injuries, global, disease burden, GBD

## Abstract

**Background:**

Road injuries remain a critical global public health issue, with spinal injuries representing a major contributor to long-term disability. This study systematically analyzes the global, regional, and national burden of spinal injuries attributable to road injuries from 1990 to 2021 and projects trends to 2046.

**Methods:**

Using data from the Global Burden of Disease (GBD) 2021 study, we estimated incidence, prevalence, and years lived with disability (YLDs) for spinal injuries caused by road traffic, analyses spanned 204 countries, stratified by sex, age, and Socio-demographic Index (SDI) regions, GBD regions, and countries. Age-standardized rates (ASRs) per 100,000 population were calculated, and temporal trends (1990–2021) were assessed via estimated annual percentage change (EAPC). Future projections (2022–2046) utilized age-period-cohort (APC) modeling.

**Results:**

In 2021, road injuries caused 95,734 (95% UI: 66,597–138,308) incident spinal injuries globally, with 2.63 million (95% UI: 2.39–2.93 million) prevalent cases and 777,365 (95% UI: 552,847–1,004,818) YLDs. Males bore 2.9–2.7 times higher burden than females. Age-standardized incidence (ASIR), prevalence (ASPR), and YLDs rates (ASYLDR) peaked in the 65–69 age group (ASPR: 75.00/100,000; ASYLDR: 20.64/100,000). High SDI regions exhibited the highest ASRs (ASIR: 2.28/100,000; ASYLDR: 16.83/100,000), while Middle SDI regions had the largest absolute caseloads (incidence: 27,086; prevalence: 703,112). From 1990 to 2021, global ASIR declined by 40% (1.95–1.17/100,000) and ASYLDR by 46% (16.85–9.17/100,000). By 2046, male incidence is projected to rise by 5.3%, with ASYLDR remaining three-fold higher in males than females (10.86 vs. 3.61/100,000).

**Conclusion:**

Despite declining age-standardized rates, the absolute burden of road injury-related spinal trauma persists, particularly in Middle SDI regions. Targeted interventions, enhanced road safety policies, gender-specific prevention programs, and improved trauma care, are critical to mitigate disparities. Projections underscore the urgent need for equitable strategies to address rising burdens in aging and rapidly motorizing populations.

## 1 Introduction

Road injuries constitute a critical and evolving global public health challenge, disproportionately affecting populations across socioeconomic gradients. The World Health Organization (WHO) reported that in 2023, approximately 1.4 million individuals lost their lives due to road injuries, with an estimated 50 million sustaining non-fatal injuries that often result in long-term disability or functional impairment (1). Among these injuries, spinal injuries-encompassing traumatic damage to the vertebral column, spinal cord, and surrounding soft tissues-represent a subset of profound clinical and societal impact. These injuries frequently lead to permanent motor or sensory deficits, autonomic dysfunction, and reduced quality of life, imposing substantial economic burdens through acute medical care, rehabilitation, and long-term disability support, particularly in low- and middle-income countries (LMICs) where healthcare infrastructure remains strained (2, 3).

The pathogenesis of spinal injuries attributable to road injuries is multifaceted, involving mechanical forces from direct impacts, rapid deceleration, or secondary trauma due to inadequate occupant protection (lack of seatbelts, helmets, or child restraint systems). Epidemiological patterns of road injuries exhibit marked regional heterogeneity, influenced by factors such as road user composition (high prevalence of motorcycle-related injuries in Southeast Asia vs. pedestrian collisions in urbanized high-income settings), demographic profiles (young male dominance in LMICs vs. increasing older adults vulnerability in aging societies), and the effectiveness of preventive policies (mandatory helmet laws in Brazil vs. intelligent speed assistance in European Union nations) (4, 5). Despite this complexity, current global evidence on the burden of road injuries remains fragmented. Most studies focus on traumatic spinal cord injury, neglecting less severe but more prevalent vertebral injuries (fractures, dislocations) that contribute significantly to long-term disability (6). Additionally, population-based estimates of incidence, prevalence, and years lived with disability (YLDs) are often limited to specific regions or time periods, hindering the development of targeted prevention and care strategies (7).

The Global Burden of Disease (GBD) study has advanced our understanding of injury burdens worldwide, yet spinal injuries are typically aggregated within broader trauma categories (“trauma to the head, neck, and trunk”), precluding precise attribution to road traffic mechanisms (8). This limitation is critical, as road traffic accounts for an estimated 30%−40% of traumatic SI etiologies in many regions (9). Furthermore, no comprehensive analysis has projected the future burden of road injuries through 2046, a period marked by anticipated growth in motorization (especially in LMICs), urbanization, and changing travel behaviors-factors poised to exacerbate injury risks without intervention (10). Accurate projections of burdens are essential for informing healthcare resource allocation, guiding the design of vehicle safety innovations (automated emergency braking systems), and advocating for policy interventions (stricter speed limits, improved post-crash care systems) (11).

This study addresses these evidence gaps by conducting a systematic analysis of road injuries from 1990 to 2021, with projections to 2046. Leveraging data from the GBD 2021 dataset, national trauma registries, and peer-reviewed literature, we estimate incidence, prevalence, and YLDs for spinal injuries attributable to road traffic. By quantifying historical trends and modeling future trajectories, our findings aim to provide a robust evidence base for stakeholders to prioritize interventions that mitigate the escalating burden of road injuries and improve health outcomes globally.

## 2 Methods

### 2.1. Data sources

We derived data from the GBD Study 2021, the most comprehensive epidemiological dataset (12). Specifically, we accessed injury-specific estimates from the GBD Injury Module, which includes incidence, prevalence, and disability weights for spinal injuries (ICD-10 codes: S12–S13, S22–S23, S32–S33 for vertebral injuries; S14.1, S24.1, S34.1 for spinal cord injuries) attributable to road traffic (external cause code: V01–V99) (13, 14). Data spanned 204 countries/territories, 50 GBD regions, five Socio-demographic Index (SDI) regions, both sexes, and 5-year-old intervals age population from 1990 to 2021.

### 2.2 Variable definition

Incidence: number of new spinal injuries attributable to road injuries cases annually, stratified by sex (male/female), 5-year age groups (0–4 to 95+ years), 5 SDI (combining per-capita income, education, and life expectancy) regions, 50 GBD regions, and countries (15).

Prevalence: number of existing spinal injuries attributable to road injuries cases at a given time point, reflecting both incident cases and survival of prior cases.

YLDs: calculated as prevalence × disability weight, measuring the burden of non-fatal disability (16).

Age-Standardized Rates (ASRs): rates adjusted to the 2000 World Standard Population to facilitate cross-sectional comparisons, minimizing confounding by age structure differences (17).

### 2.3 Statistical analysis

#### 2.3.1 Disease burden description

We described global, regional, and national estimates of incidence, prevalence, and YLDs for 2021, presenting absolute numbers and ASRs (per 100,000 population). Stratification by sex, age, SDI regions, GBD regions, and countries.

#### 2.3.2 Temporal trend analysis

For the period 1990–2021, we used linear regression to identify estimated annual percentage change (EAPC) of ASRs, distinguishing phases of decreasing/increasing trends. Cluster analysis grouped GBD regions by EAPC values, visualized via dendrograms to identify shared trend patterns.

#### 2.3.3 Future burden projections

All estimates included 95% uncertainty intervals (UIs) derived from GBD's probabilistic modeling, which propagates uncertainty through data sources, modeling assumptions, and parameter estimates. Future projections (2022–2046) were generated using an age-period-cohort (APC) model within a maximum likelihood framework. This model decomposes temporal trends into three distinct components: age effects (variation in risk across age groups), period effects (variations affecting all age groups simultaneously over time, e.g., policy changes), and cohort effects (variations related to birth cohorts). Model parameters were estimated using the intrinsic estimator approach to address the inherent identifiability problem in APC models. Projections were derived by extrapolating period and cohort trends observed during 1990–2021, while holding age effects constant. Uncertainty intervals were propagated using the same probabilistic framework as the GBD study. Statistical significance was defined as *p* < 0.05. Analyses were performed in R (version 4.2.3), using dplyr for data manipulation, ggplot2 for visualization, and domain-specific packages for statistical modeling (such as BAPC).

## 3 Results

### 3.1 The disease burden of spinal injuries attributable to road injuries in 2021

In 2021, the global number of spinal injuries attributable to road injuries-related incidence cases was 95,734 (95% UI: 66,597–138,308). The corresponding age-standardized incidence rate (ASIR) was 1.17 (95% UI: 0.82–1.69) per 100,000 population. The number of spinal injuries-related prevalence cases reached 2,628,507 (95% UI: 2,392,869–2,934,125), with an age-standardized prevalence rate (ASPR) of 30.95 (95% UI: 28.20–34.57) per 100,000 population. The global number of YLDs attributable to spinal injuries from road injuries was 777,365 (95% UI: 552,847–1,004,818), and the age-standardized YLDs rate (ASYLDR) was 9.17 (95% UI: 6.52–11.85) per 100,000 population (Supplementary Tables S1–S3).

Sex disparities were evident in 2021. Males consistently bore a disproportionately higher burden, exhibiting approximately 2.9 times higher incidence, 2.4 times higher prevalence, and 2.7 times higher YLDs compared to females. Males exhibited higher age-standardized rates across all metrics, with ASIR, ASPR, and ASYLDR values 2.90, 2.50, and 2.74 times greater than those in females (Supplementary Figure S1, Supplementary Tables S1–S3).

Age-stratified analysis revealed a progressive increase in burden with advancing age, peaking in the 65–69 age group. This cohort exhibited the highest age-standardized prevalence (75.00/100,000) and YLD rates (20.64/100,000; Supplementary Table S2).

At the SDI region level, the Middle SDI region demonstrated the highest incidence burden with 27,086 cases (95% UI: 18,512–39,285), followed by High SDI (26,492; 95% UI: 17,761–38,662) and High-middle SDI regions (20,285; 95% UI: 13,888–29,178). For prevalence, the High SDI region reported the largest caseload at 866,184 (95% UI: 772,653–992,314), substantially exceeding Middle SDI (703,112; 95% UI: 645,590–778,385) and High-middle SDI regions (616,448; 95% UI: 562,084–683,752). YLDs followed a similar pattern, with High SDI (236,746; 95% UI: 164,092–310,284) and Middle SDI (213,292; 95% UI: 153,190–275,344) regions bearing the greatest disability burden. ASRs revealed pronounced disparities. The High SDI region exhibited the highest ASIR of 2.28/100,000 (95% UI: 1.55–3.27), ASPR of 60.73/100,000 (95% UI: 53.88–70.30), and ASYLDR of 16.83/100,000 (95% UI: 11.58–22.16). In contrast, Low SDI regions showed the lowest rates: ASIR 0.66/100,000 (95% UI: 0.45–0.95), ASPR 13.55/100,000 (95% UI: 12.56–14.89), and ASYLDR 4.88/100,000 (95% UI: 3.56–6.15). Notably, Middle SDI regions displayed moderate ASRs (ASIR 1.04/100,000; ASPR 25.36/100,000) despite their high absolute case numbers (Supplementary Tables S1–S3, Supplementary Figure S3). These findings highlight a dual burden: higher SDI regions face elevated age-standardized rates, while middle SDI regions shoulder the largest absolute caseloads.

Across the 54 GBD regions, Asia ranked the top one in number of incidence cases (49,925), followed by Basic Health System (42,243) and Advanced Health System (33,458). Advanced Health System also ranked the top one for number of prevalence cases (1,097,251), followed by Asia (1,304,476) and Europe (442,734). For YLDs cases, Asia led (393,285), followed by Advanced Health System (301,063) and America (192,201). However, Oceania ranked the bottom one for incidence (119), prevalence (2,321), and YLDs (853). For ASRs, High-income North America had the highest ASIR (3.52/100,000), while Commonwealth Low Income ranked the lowest (0.39/100,000). The highest ASPR was in High-income North America (94.53/100,000), and the lowest in Commonwealth Low Income (8/100,000). High-income North America also led in ASYLDR (25.74/100,000), with Commonwealth Low Income again at the bottom (2.81/100,000). Regions consistently ranking low included Commonwealth Low Income, Western Africa, and Western Sub-Saharan Africa (Supplementary Figure S4, Supplementary Tables S1–S3).

**Figure 1:**
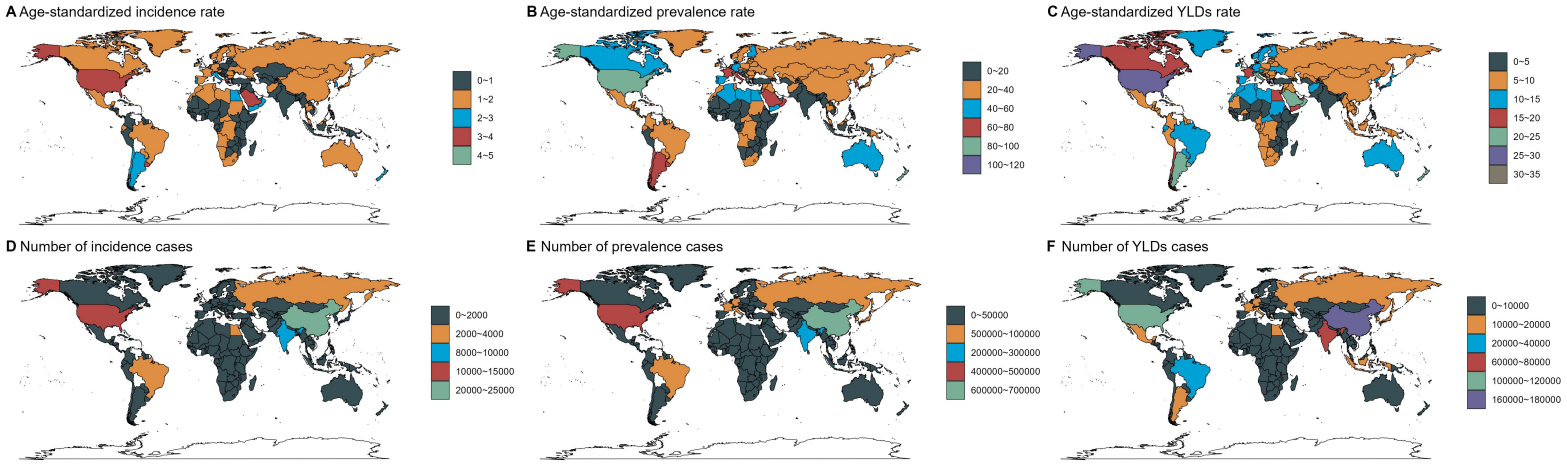
Numbers and age-standardized rates of spinal injuries attributable to road injuries-related incidence, prevalence, and YLDs across countries and territories in 2021.

Country-level variations were pronounced. Saudi Arabia, the United States, and Andorra demonstrated the highest age-standardized incidence rates, whereas Bangladesh, Ethiopia and Nigeria showed the lowest. In absolute terms, China, the United States and India reported the largest caseloads, while small island nations like Tokelau and Niue had minimal burdens (Figure 1, Supplementary Tables S1–S3).

### 3.2 Temporal trend for spinal injuries attributable to road injuries-related disease burden from 1990 to 2021

**Figure 2:**
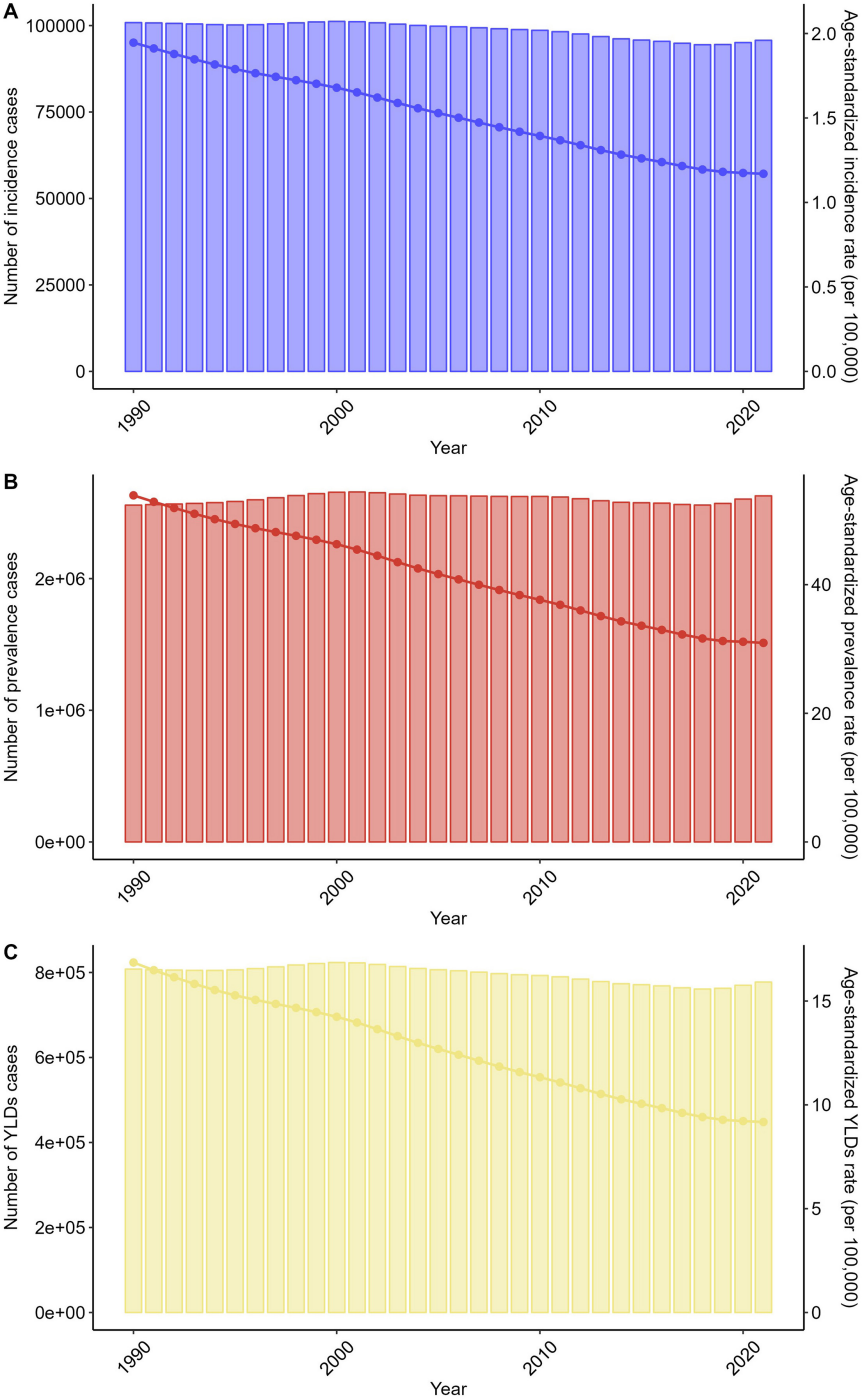
Trends in the numbers and age-standardized rates of spinal injuries attributable to road injuries-related incidence, prevalence, and YLDs globally from 1990 to 2021.

Globally, the number of spinal injuries attributable to road injuries cases remained relatively stable. The number of incidence cases changed from 100,850 (95% UI: 70,064–144,763) in 1990 to 95,734 (95% UI: 66,597–138,308) in 2021. The number of prevalence cases changed from 2,558,050 (95% UI: 2,309,837–2,915,989) to 2,628,507 (95% UI: 2,392,869–2,934,125), and the number of YLDs cases changed from 807,862 (95% UI: 576,388–1,042,770) to 777,365 (95% UI: 552,847–1,004,818). Regarding the corresponding ASRs, they all changed in an downward direction. The ASIR decreased from 1.95 (95% UI: 1.35–2.79) to 1.17 (95% UI: 0.82–1.69), the ASPR decreased from 53.88 (95% UI: 48.6–61.14) to 30.95 (95% UI: 28.2–34.57), and the ASYLDR decreased from 16.85 (95% UI: 12.03–21.7) to 9.17 (95% UI: 6.52–11.85) per 100,000 population (Figure 2, Supplementary Tables S1–S3).

The trends in males and females separately were consistent with those of the overall population (Supplementary Figure S5, Supplementary Tables S1–S3). Additionally, the trends were consistent across all age groups (Supplementary Figure S6, Supplementary Tables S1–S3). At the SDI region level, all SDI regions demonstrated the same trend as the overall population (Supplementary Figure S7, Supplementary Tables S1–S3).

**Figure 3:**
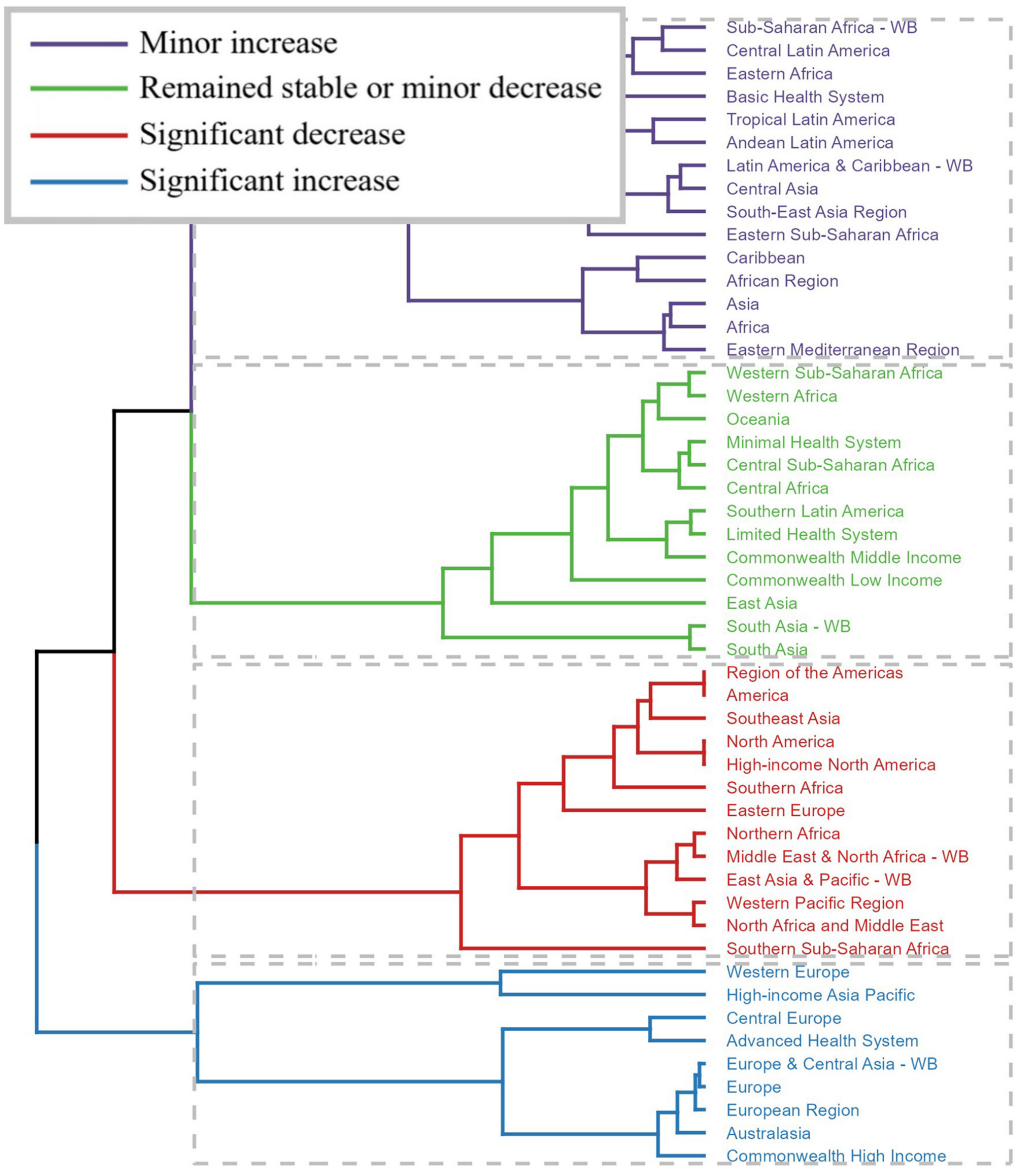
Results of cluster analysis based on the EAPC values of the spinal injuries attributable to road injuries-related age-standardized rates for incidence, prevalence, and YLDs from 1990 to 2021.

Across GBD regions, the trend of the spinal injuries attributable to road injuries-related disease burden showed variability. The results of cluster analysis are presented in Figure 3. A significant increase in incidence, prevalence, and YLDs rate occurred in Western Europe, High-income Asia Pacific, Central Europe, Advanced Health System, Europe and Central Asia -WB, Europe, European Region, Australasia, and Commonwealth High Income. In contrast, a significant decrease was observed in Region of the Americas, America, Southeast Asia, North America, High-income North America, Southern Africa, Eastern Europe, Northern Africa, Middle East and North Africa—WB, East Asia and Pacific-WB, Western Pacific Region, North Africa and Middle East, and Southern Sub-Saharan Africa (Figure 3).

**Figure 4:**
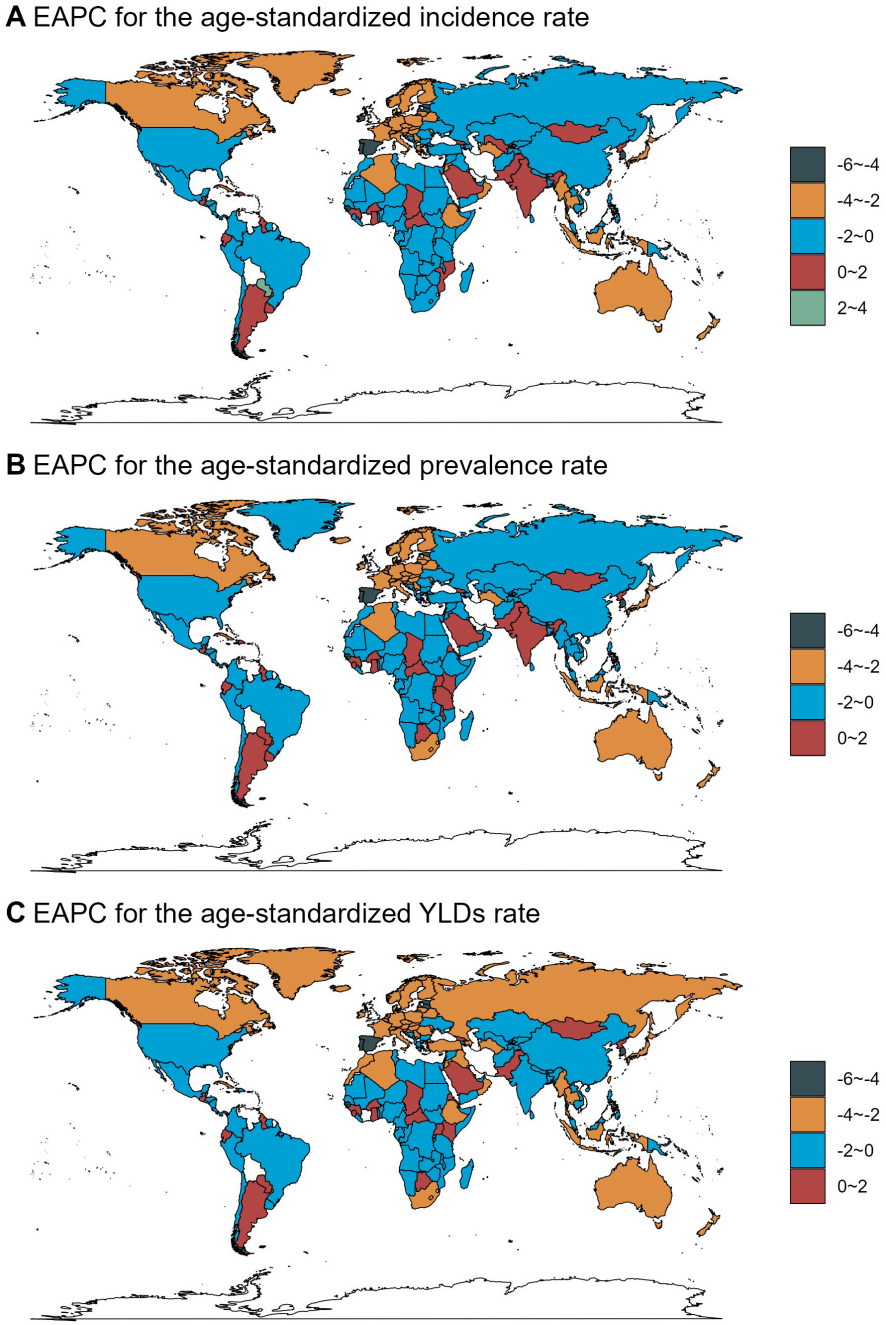
The EAPC of spinal injuries attributable to road injuries-related ASRs from 1990 to 2021.

Across countries and territories, the changing trend also differed. From 1990 to 2021, Paraguay exhibited the most significant increase in ASIR [EAPC = 2.02, 95% confidence interval (CI): 1.88–2.17], ASPR (EAPC = 1.84, 95% CI: 1.72–1.96), and ASYLDR (EAPC = 1.56, 95% CI: 1.43–1.68). Estonia showed the most significant decrease in ASIR (EAPC = −5.35, 95% CI: −5.65 to −5.04) and ASYLDR (EAPC = −4.91, 95% CI: −5.17 to −4.64). Portugal showed the most significant decrease in ASPR (EAPC = −4.61, 95% CI: −4.86 to −4.37; Figure 4, Supplementary Tables S1–S3).

### 3.3 The predicted results from 2022 to 2046

**Figure 5:**
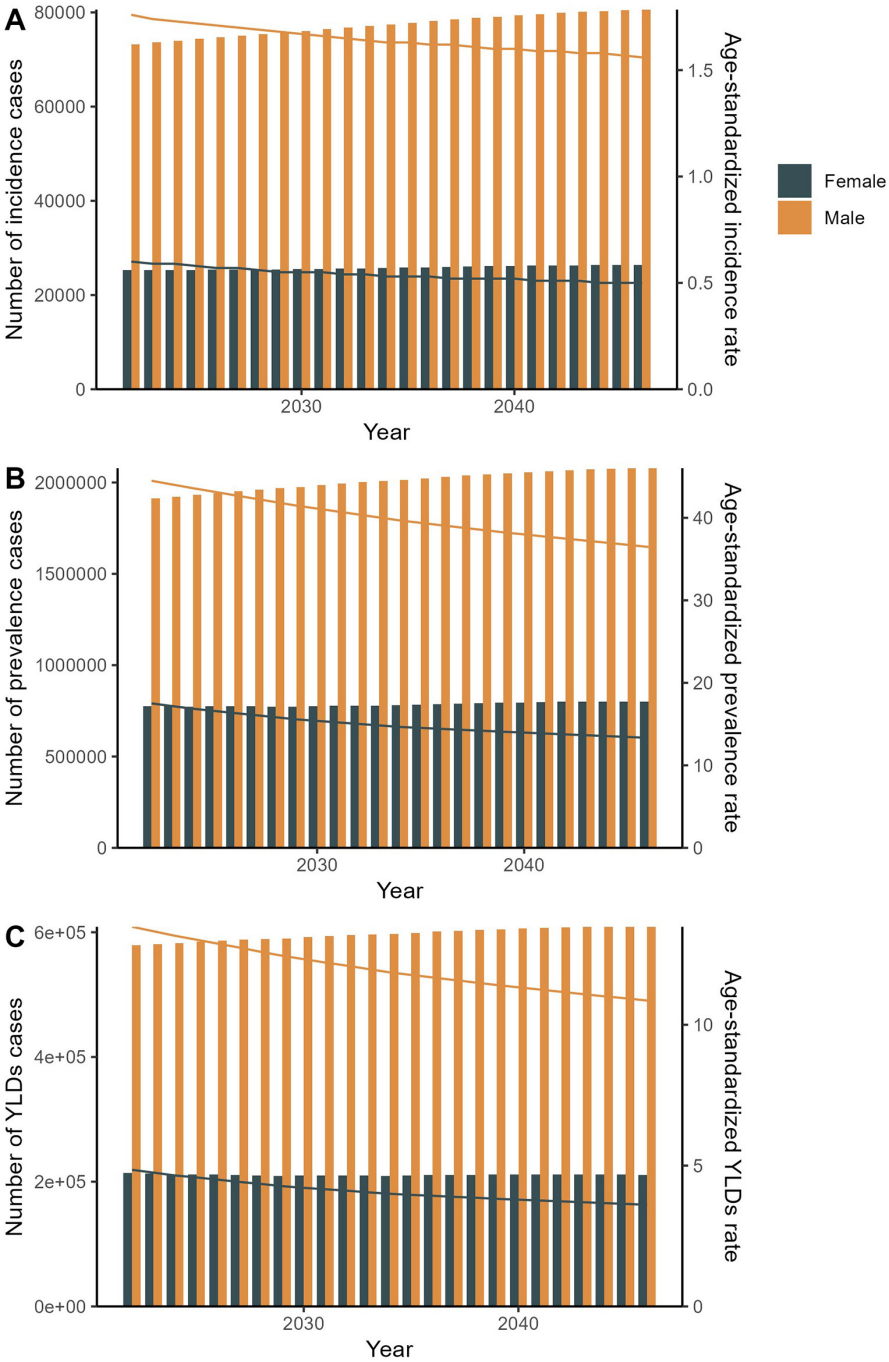
The predicted results in the spinal injuries attributable to road injuries-related numbers and age-standardized rates of incidence, prevalence, and YLDs by sex globally from 2022 to 2046 of the APC model.

The predicted results of the data indicated that the number of incidence, prevalence, and YLDs cases for both genders would show certain trends from 2022 to 2046. For males, referring to the data from the provided table, the number of incidence cases was 73,223.37 in 2022 and was predicted to be 80,597.74 in 2046. The number of prevalence cases was 1,912,514.32 in 2022 and was projected to reach 2,078,419.51 in 2046. The number of YLDs was 579,162.87 in 2022 and was expected to be 608,711.78 in 2046. For females, the number of incidence cases would increase from 25,265.03 in 2022 to 26,385.51 in 2046. The number of prevalence cases would increase from 776,500.03 to 801,406.13 during this period. The number of YLDs cases would decrease from 213,706.04 to 211,099.03. Moreover, the predicted results showed that the corresponding ASRs would also change over the next 25 years for both genders. In 2022, the ASIR for males was 1.76 per 100,000 population and was predicted to be 1.56 in 2046. The ASPR for males was 44.49 in 2022 and was projected to be 36.47 in 2046. During the same period, the ASYLDR decreased from 13.48 to 10.86. For females, the ASIR would decrease from 0.60 to 0.50, the ASPR would decrease from 17.51 to 13.33, and the ASYLDR would decrease from 4.85 to 3.61 (Figure 5, Supplementary Table S4).

## 4 Discussion

This comprehensive analysis quantifies the substantial and evolving global burden of spinal injuries attributable to road injuries, revealing critical disparities across demographics, geographies, and socioeconomic strata. In 2021, road injuries caused 95,734 incident spinal injury cases globally, contributing to 2.63 million prevalent cases and 777,365 YLDs. The disproportionate burden on males, incidence, prevalence, and YLDs rates 2.9–2.7 times higher than females, aligns with global patterns of male-dominated risk-taking behaviors, occupational exposures, and higher road traffic participation (18). Age-stratified trends highlighted a progressive rise in burden peaking in the 65–69 age group, likely reflecting cumulative exposure to road trauma, delayed degenerative complications, and age-related declines in physiological resilience (19). Geographically, while High SDI regions exhibited the highest age-standardized rates (ASIR: 2.28/100,000; ASYLDR: 16.83/100,000), Middle SDI regions bore the largest absolute caseloads, underscoring the dual challenge of aging populations in high-income nations and expanding motorization with inadequate safety infrastructure in middle-income regions (20). Temporal trends from 1990 to 2021 revealed a 40% decline in global ASIR and a 46% reduction in YLDs rates, likely reflecting advancements in road safety policies, vehicle design, and acute trauma care (21). However, projections to 2046 suggest persistent inequalities, with rising absolute cases in aging populations despite declining ASRs.

The pronounced male predominance in spinal injury burden (2.9–2.7 times higher than females) likely reflects globally observed patterns of male-dominated risk-taking behaviors (e.g., speeding, alcohol-impaired driving), greater occupational exposure (e.g., commercial driving), and higher utilization of motorcycles, particularly in Middle SDI regions where motorcycle-related trauma is prevalent (22). For instance, in Middle SDI regions, where motorcycle-related injuries dominate road trauma, males accounted for >70% of spinal injury YLDs, consistent with studies from Southeast Asia and Sub-Saharan Africa (23). Conversely, the lower female burden may reflect reduced mobility in patriarchal societies, though underdiagnosis due to healthcare access barriers cannot be excluded (24).

Age-related trends further emphasize the interplay of exposure and vulnerability. The steep rise in prevalence and YLDs among older adults (65–69 years: ASPR 75.00/100,000) aligns with evidence linking spinal injuries in this cohort to frailty, osteoporosis, and prolonged recovery times (25). In High SDI regions, aging populations with high vehicle ownership face elevated risks of low-impact spinal trauma (whiplash), while in Low SDI regions, older adults remain vulnerable due to pedestrian injuries and limited post-crash care (26).

Geographic disparities reflect divergent developmental trajectories. High SDI regions, such as High-income North America (ASYLDR: 25.74/100,000), combine high vehicle density with aging populations, amplifying per capita disability burdens. The concentration of the largest absolute caseloads in Middle SDI regions (e.g., China, India), despite their moderate age-standardized rates (ASIR 1.04/100,000; ASPR 25.36/100,000), highlights the “injury transition” phenomenon (27). Rapid motorization and urbanization in these regions have outpaced the implementation and enforcement of comprehensive road safety regulations and infrastructure improvements. This has led to a surge in road traffic exposure and injury incidence within large populations, driving high absolute numbers even as age-standardized rates decline. This contrasts with High SDI regions, where elevated ASRs primarily reflect aging populations and high vehicle density. Meanwhile, Low SDI regions (Bangladesh, Ethiopia) report minimal age-standardized rates (ASIR < 0.5/100,000), likely reflecting underreporting, limited diagnostic capacity, and lower vehicle ownership rather than true risk reduction (28).

The global decline in age-standardized rates (ASIR: 1.95–1.17/100,000; 1990–2021) coincides with WHO-endorsed road safety initiatives, including seatbelt/helmet laws, trauma system development, and safer road designs (29). For example, Estonia's 5.35% annual ASIR decline aligns with its stringent alcohol control policies and adoption of EU vehicle safety standards (30). Conversely, Paraguay's 2.02% annual ASIR increase mirrors Latin America's struggles with unregulated motorcycle use and underinvestment in trauma care (31).

Notably, High SDI regions demonstrated diverging trends: while Western Europe saw rising burdens (aging-related spinal degeneration), High-income North America reduced ASYLDR by 22% (1990–2021), likely due to advanced prehospital care and spinal stabilization protocols (32). In contrast, Low-middle SDI regions in Sub-Saharan Africa showed stagnant trends, reflecting systemic gaps in injury surveillance and rehabilitation access (33).

Projections indicate a 5.3% increase in male incidence cases (2022–2046) despite declining ASIR, driven by population growth and aging. For females, stable incidence but rising prevalence (801,406 cases by 2046) suggests improved survival with chronic disability, necessitating expanded rehabilitation services (34). The persistent three-fold male-female ASYLDR necessitates occupational safety programs targeting male-dominated high-risk industries (e.g., trucking, construction) (35).

Regionally, Middle SDI nations require urgent scaling of helmet/seatbelt enforcement and investment in trauma systems to manage the “dual burden” of rising absolute cases from motorization and persistent high rates from lagging policies. For example, India's projected 170,218 YLDs by 2046 highlights urgent needs for motorcycle helmet enforcement and spinal injury registries (36). Conversely, High SDI regions must prioritize mitigating aging-related vulnerability through fall prevention programs for older pedestrians and integrating adaptive vehicle safety technologies (e.g., autonomous emergency braking systems optimized for older adults occupants) (37).

This study has several limitations. First, GBD data rely on modeled estimates, potentially underestimating burdens in regions with sparse injury registries (Sub-Saharan Africa) (38). Second, disability weights assume uniform health-state valuations, though cultural differences in pain perception and functional limitations may skew YLD calculations (39). Third, projections exclude disruptive technologies (autonomous vehicles) that could alter injury patterns by 2046 (40). Finally, the analysis does not capture socioeconomic gradients within nations, masking disparities between urban and rural populations (41).

## 5 Conclusion

This study underscores road injury-related spinal trauma as a persistent global health challenge, marked by inequities across sex, age, and development strata. While declining age-standardized rates reflect decades of road safety progress, rising absolute burdens in Middle SDI regions demand urgent action. Prioritizing helmet/seatbelt enforcement, trauma system strengthening, and rehabilitation access in motorizing nations could avert millions of disability-adjusted life years by 2046. Future research must address surveillance gaps in Low SDI regions and evaluate equity-focused interventions to ensure progress aligns with the WHO's Decade of Action for Road Safety.

## Data Availability

The original contributions presented in the study are included in the article/Supplementary material, further inquiries can be directed to the corresponding authors.
