# Supplementary material for: Global, regional, and national burden of spinal injuries attributable to road injuries: a systematic analysis of incidence, prevalence, and YLDs with projections to 2046
Source: Front Public Health. 2025 Sep 18;13:1628455. doi: 10.3389/fpubh.2025.1628455 (PMC12488732; doi:10.3389/fpubh.2025.1628455)

**Supplementary Information and Data**

# Global, regional, and national burden of spinal injuries attributable to road injuries: a systematic analysis of incidence, prevalence, and YLDs with projections to 2046

## Supplementary Figures

**Figure S1.** Numbers and age-standardized rates of spinal injuries attributable to road injuries related incidence, prevalence, and YLDs for both sex in 2021.

**Figure S2.** Numbers and age-standardized rates of spinal injuries attributable to road injuries related incidence, prevalence, and YLDs for different age groups in 2021.

**Figure S3.** Numbers and age-standardized rates of spinal injuries attributable to road injuries related incidence, prevalence, and YLDs for different SDI region in 2021.

**Figure S4.** Numbers and age-standardized rates of spinal injuries attributable to road injuries related incidence, prevalence, and YLDs for different GBD region in 2021.

**Figure S5.** Trends in the numbers and age-standardized rates of spinal injuries attributable to road injuries-related incidence, prevalence, and YLDs globally by sex from 1990 to 2021.

**Figure S6.** Trends in the numbers and age-standardized rates of spinal injuries attributable to road injuries-related incidence, prevalence, and YLDs globally by age groups from 1990 to 2021.

**Figure S7.** Trends in the numbers and age-standardized rates of spinal injuries attributable to road injuries-related incidence, prevalence, and YLDs globally by SDI region from 1990 to 2021.

**Figure S1.** Numbers and age-standardized rates of spinal injuries attributable to road injuries related incidence, prevalence, and YLDs for both sex in 2021.

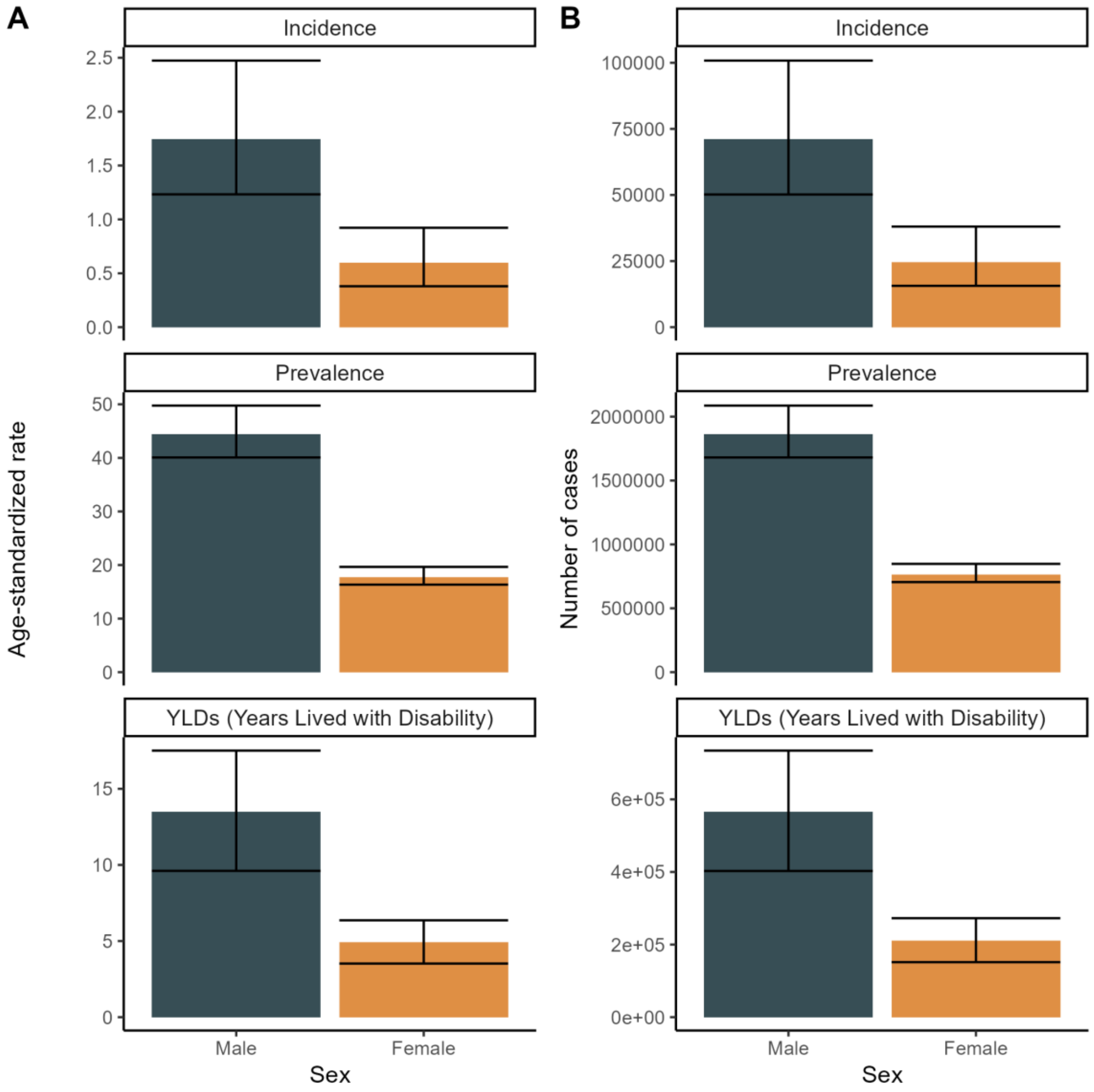

**Figure S2.** Numbers and age-standardized rates of spinal injuries attributable to road injuries related incidence, prevalence, and YLDs for different age groups in 2021.

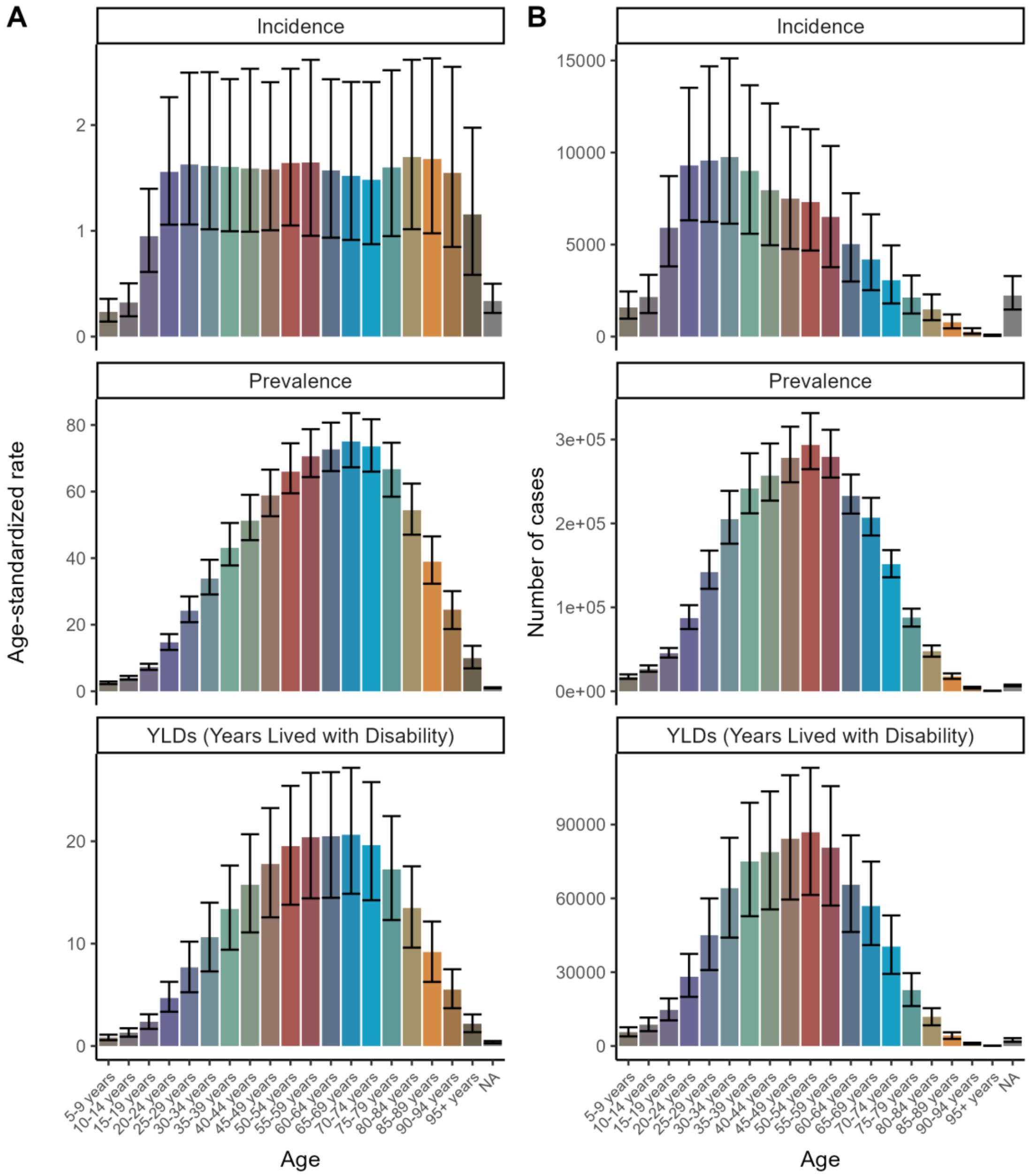

**Figure S3.** Numbers and age-standardized rates of spinal injuries attributable to road injuries related incidence, prevalence, and YLDs for different SDI region in 2021.

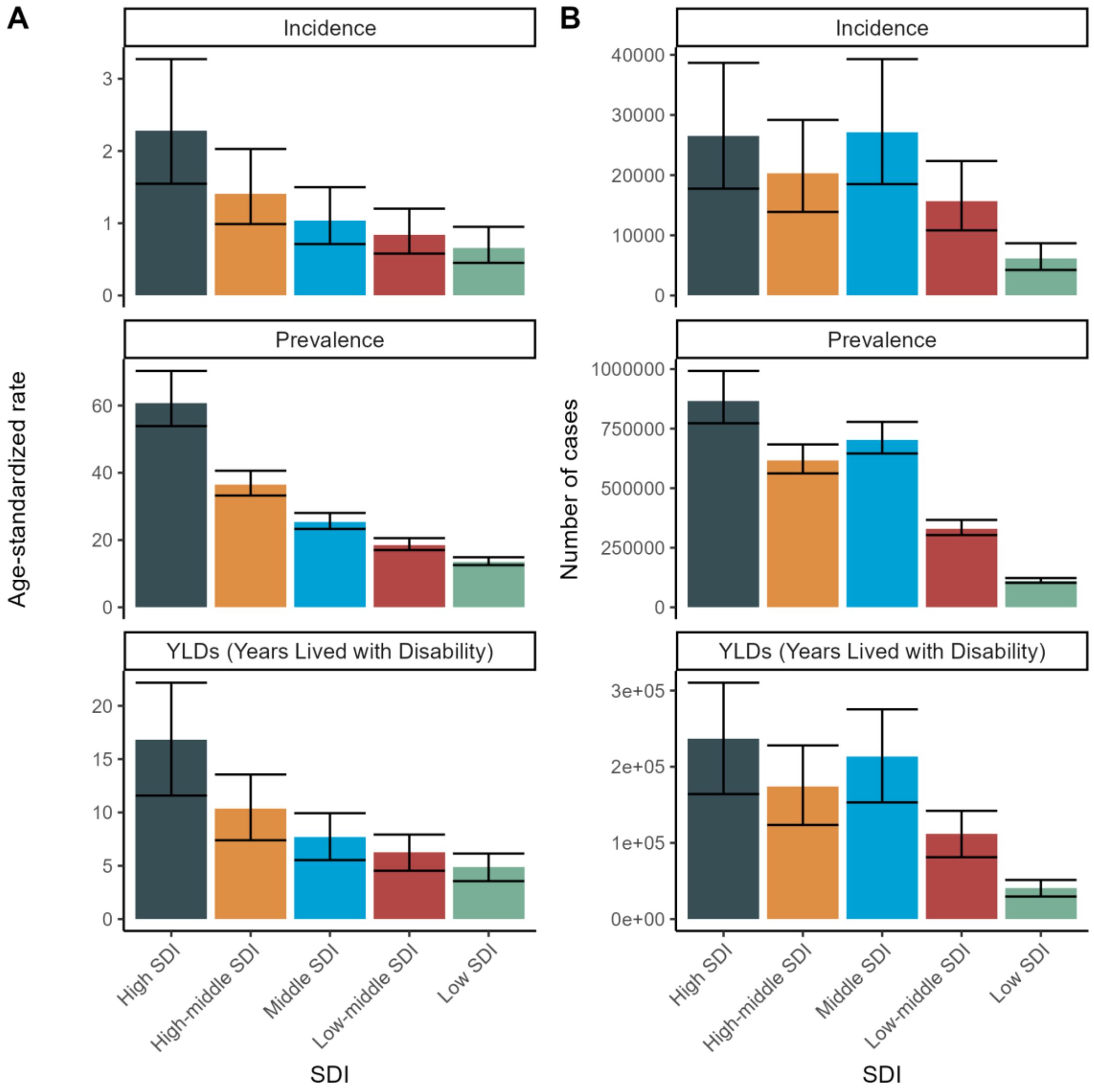

**Figure S4.** Numbers and age-standardized rates of spinal injuries attributable to road injuries related incidence, prevalence, and YLDs for different GBD region in 2021.

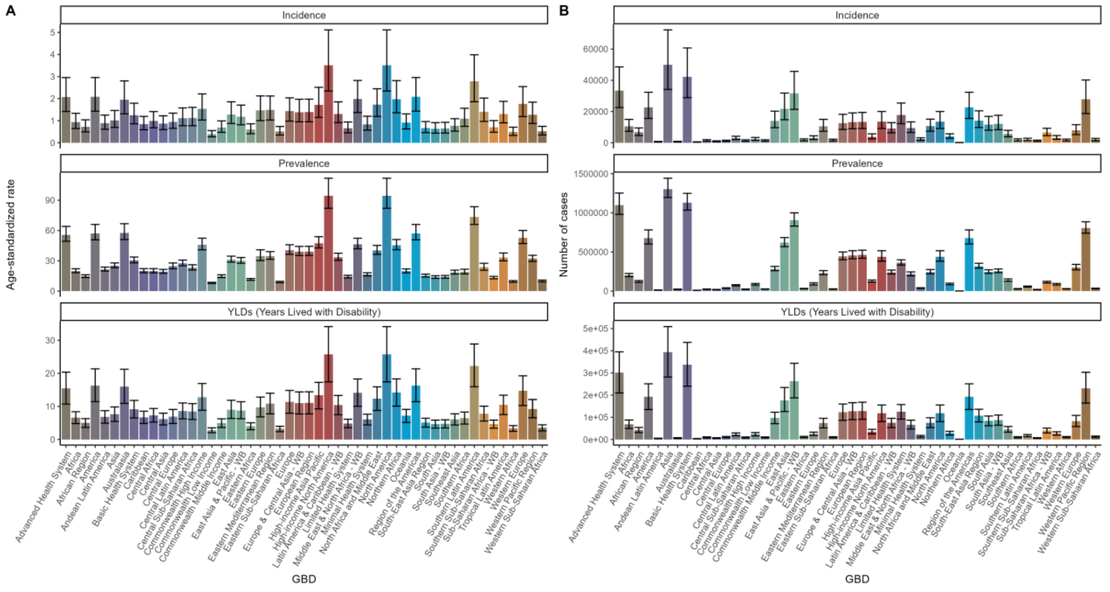

**Figure S5.** Trends in the numbers and age-standardized rates of spinal injuries attributable to road injuries-related incidence, prevalence, and YLDs globally by sex from 1990 to 2021.

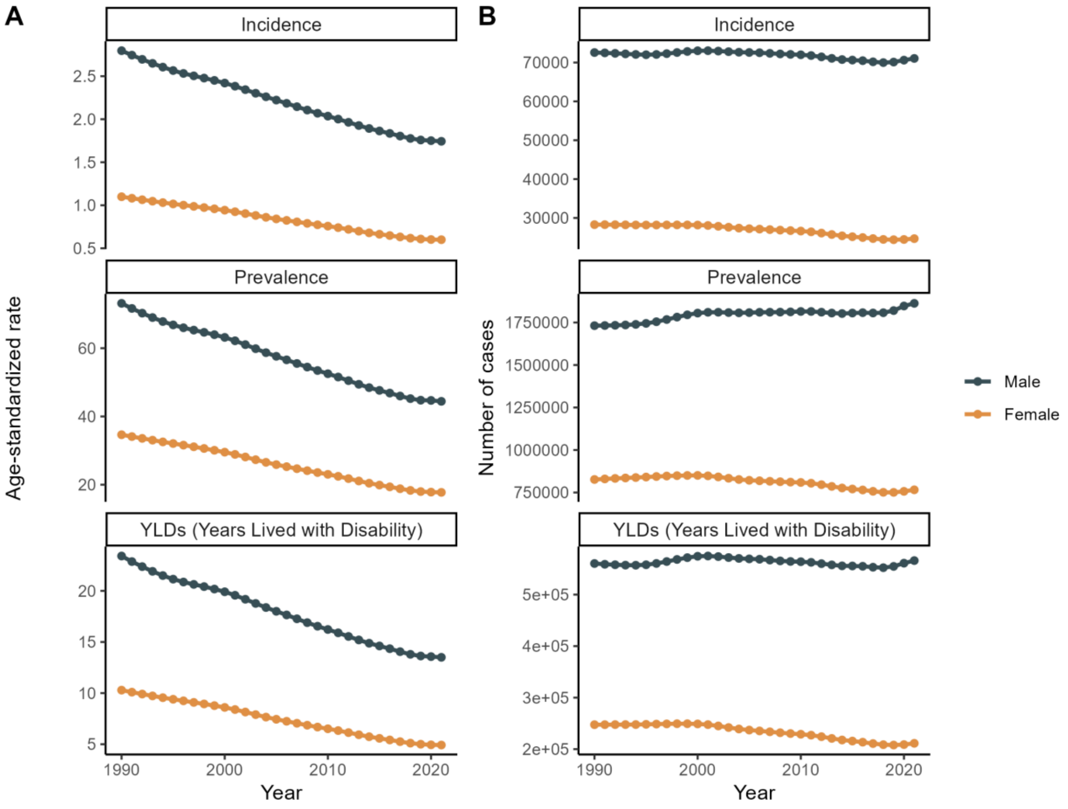

**Figure S6.** Trends in the numbers and age-standardized rates of spinal injuries attributable to road injuries-related incidence, prevalence, and YLDs globally by age groups from 1990 to 2021.

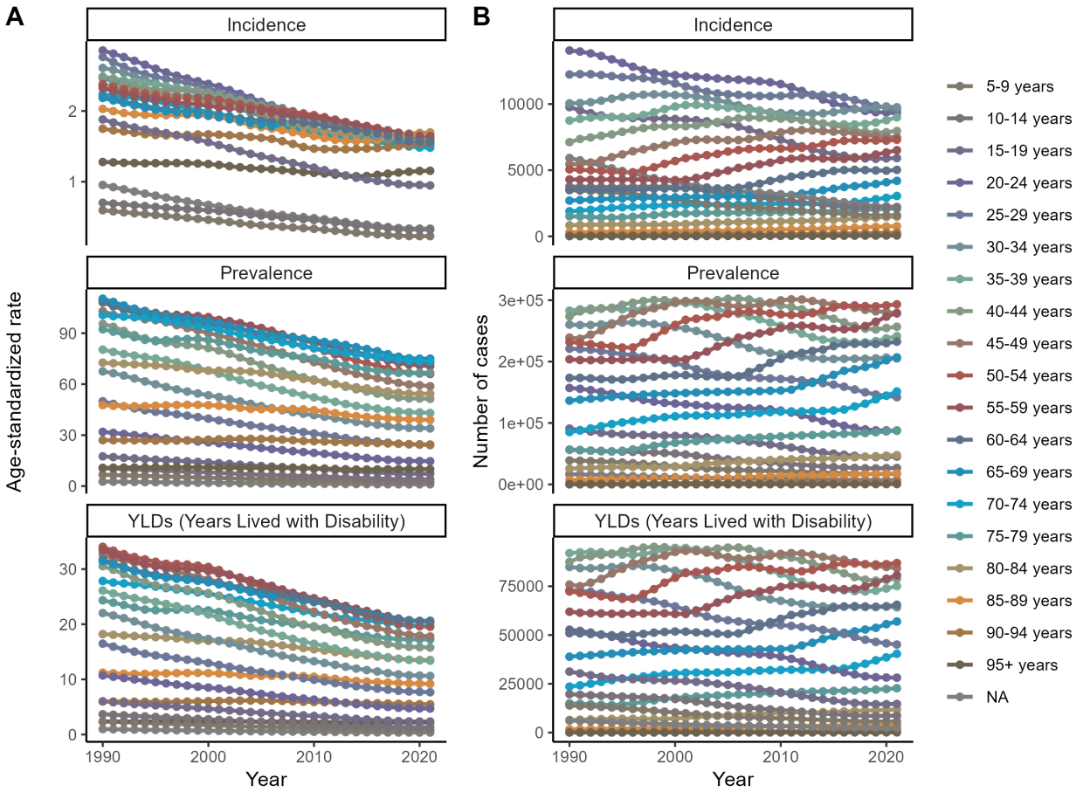

**Figure S7.** Trends in the numbers and age-standardized rates of spinal injuries attributable to road injuries-related incidence, prevalence, and YLDs globally by SDI region from 1990 to 2021.

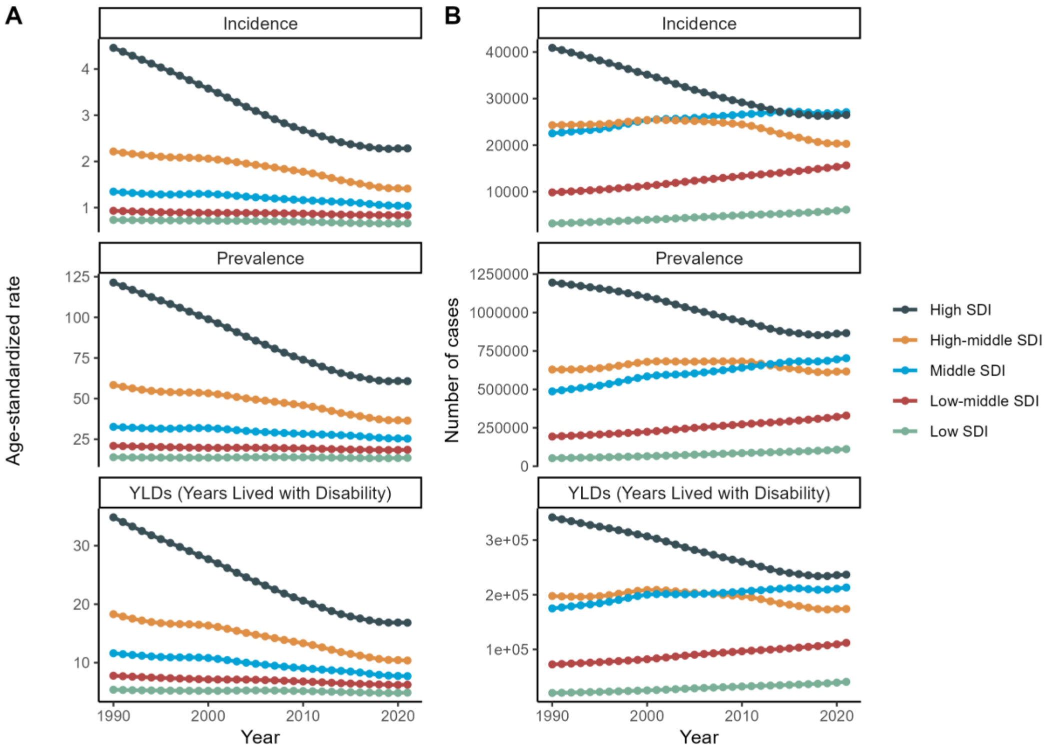

Supplement: Supplementary file 1 [file Data_Sheet_1.pdf]
