# Supplementary material for: Global, regional, and national burden of spinal injuries attributable to road injuries: a systematic analysis of incidence, prevalence, and YLDs with projections to 2046
Source: Front Public Health. 2025 Sep 18;13:1628455. doi: 10.3389/fpubh.2025.1628455 (PMC12488732; doi:10.3389/fpubh.2025.1628455)
Supplement: Supplementary file 2 [file Supplementary_file_1.docx]

**Table legend**

**Table 1** The number of incidence cases and the age-standardized incidence rate attributable to spinal injuries attributable to road injuries in 1990 and 2021, and its trends from 1990 to 2021 globally.

**Table 2** The number of prevalence cases and the age-standardized prevalence rate attributable to spinal injuries attributable to road injuries in 1990 and 2021, and its trends from 1990 to 2021 globally.

**Table 3** The number of YLDs cases and the age-standardized YLDs rate attributable to spinal injuries attributable to road injuries in 1990 and 2021, and its trends from 1990 to 2021 globally.

**Table 4** The predicted results in the spinal injuries attributable to road injuries-related numbers and age-standardized rates of incidence, prevalence, and YLDs by sex globally from 2022 to 2046 of the APC model.
